# Suicidal thoughts, problem gambling severity and utilisation of health care and social services: A population-based study in Finland

**DOI:** 10.1016/j.abrep.2025.100658

**Published:** 2025-12-19

**Authors:** Tiina Latvala, Maria Heiskanen, Virve Marionneau, Kalle Lind, Tanja Grönroos, Sari Castrén

**Affiliations:** a Finnish Institute for Health and Welfare, Department of Promotional and Preventive Work, P.O. Box 30, FI-00271 Helsinki, Finland; b University of Helsinki, Faculty of Social Sciences, Centre for Research on Addiction, Control and Governance, Finland; c University of Helsinki, Faculty of Social Sciences, P.O. Box 54, FI-00014 Helsinki, Finland; d Social Sciences Department of Psychology and Speech-Language Pathology Turku, University of Turku, Turku, Finland; e Department of Medicine, University of Helsinki, Helsinki, Finland

**Keywords:** Gambling, Suicidal thoughts, Utilization of health care, Utilization of social services

## Abstract

**Background:**

Persons harmed by problem gambling have an increased risk of suicide and suicidal thoughts. Social and health care services have an important role in recognising and supporting persons harmed by gambling problems and suicidal thoughts.

**Methods:**

We analyse population-based survey data from the Healthy Finland survey of permanent Finnish residents aged 20 years or older (n = 28,154). We study associations between gambling and suicidal thoughts, and use of health care and social services among individuals engaged in gambling and experienced suicidal thoughts in the past year. The analysis uses χ^2^ tests and logistic regression models.

**Results:**

Of persons harmed by problem gambling (PGSI score ≥ 8) 31 % had suicidal thoughts. Among persons harmed by problem gambling and having suicidal thoughts, utilization of health care and social services was more common compared to non-gamblers with suicidal thoughts. Individuals who engaged in gambling and experienced suicidal thoughts in the past year but had not utilized health care services were more often young, male, and excessive alcohol users. On the contrary, those who had used social services, engaged in gambling and had suicidal thoughts were experiencing more often long-term illnesses and severe psychological distress.

**Conclusions:**

Suicidal thoughts are prevalent among persons harmed by problem gambling. Social and health service systems need to better recognise the association between gambling and co-occurrent suicidal thoughts. In addition, services need to be developed to offer effective treatment and support with high levels of integration.

## Highlights

• Suicidal thoughts are common among people with problem gambling, even after accounting for other confounding factors.
• Of individuals with both problem gambling and suicidal thoughts 96% had used health care and 37% social services in the past year.
• Those with both suicidal thoughts and problem gambling were more likely to use services than those with suicidal thoughts alone.
• Gamblers with suicidal thoughts who had not used health services were more often young, male, and excessive alcohol users.
• Improved screening, early detection, and better integration of services are needed to address gambling problems and suicidality.

## 1 Introduction

Gambling is associated with wide-ranging negative consequences to public health. Suicidality is one of the most severe gambling-related harms. Suicidality refers to a range of self-harm, including suicidal ideations and thoughts, suicidal behaviour, suicide attempts and completed suicide (Nock et al., 2008). Suicide attempts involve intentional self-harm without fatal outcomes (Hawton et al., 2012), while suicidal ideation can range from passive thoughts to active plans (Klonsky et al., 2016). As discussed by O’Carroll et al. (1996) and later Silverman et al. (2007), the term *suicidality* covers a broad continuum of thoughts and behaviours that vary in intent and outcome. Suicide denotes self-inflicted death with intent to die, whereas suicide attempts involve nonfatal self-harm with some degree of intent. Suicidal acts encompass such behaviours regardless of injury, while instrumental suicide-related behaviours mimic suicidal intent without a wish to die. Together, these constitute suicide-related behaviour. It is also important to distinguish between ideation—purely cognitive thoughts about suicide—and intent, which reflects stronger engagement and a desire to act, with planning representing a further step. Although impulsivity may disrupt this sequence, ideation and intent remain central and dynamic. This study focuses on suicidal thoughts, defined here as a synonym of suicidal ideation.

Prior research shows an association between gambling and the full spectrum of suicidality: Review studies show that persons harmed by gambling problems have an increased risk of suicide and suicidal ideations (Andreeva et al., 2022, Kristensen et al., 2024, Wang et al., 2025). In population samples, persons harmed by problem gambling report higher levels of suicidal ideations and suicide attempts than persons non-harmed by gambling problems (e.g., Sundqvist and Wennberg, 2022, Wardle et al., 2020, 2023). A meta-analysis by Kristensen et al. (2024) showed that 31.6 % of persons harmed by problem gambling experienced lifetime suicidal ideation, and 13.2 % had lifetime suicide attempts. Association between gambling and suicidality was also confirmed by recent *meta*-analysis which discovered that, among persons harmed by gambling problem, odds for suicidal ideation (OR = 1.58), suicide attempts (OR = 2.91) and suicide mortality (OR = 8.52) were significantly higher than for persons non-harmed by gambling problems (Wang et al., 2025). Further, this study also indicated that strength of these associations may vary across populations and contexts.

The association between gambling and suicidality is complex and can be partly explained by underlying psychiatric comorbidities, mood disorders, substance use disorders, trauma, or personality characteristics (Guillou-Landreat et al., 2016, Håkansson and Karlsson, 2020). However, increasing evidence connects gambling directly to suicidality (also Wardle et al., 2024). A review of qualitative evidence showed that in most cases, gambling precedes other risk factors for suicidality (Marionneau & Nikkinen, 2022). Associations between gambling and suicidal ideations have persisted in population studies even after adjusting for confounding factors such as mental health disorders, substance use and depression (Kristensen et al., 2024, Sundqvist and Wennberg, 2022, Wardle et al., 2020). Longitudinal population study evidence shows that increases in problem gambling severity are associated with increases in suicide attempts over time (Wardle et al., 2023).

### 1.1 Treatment for gambling-related suicidality

Treatment services for gambling harms need to be well-equipped to respond to suicidality. Research has reported high levels of suicidality among problem gambling help-seekers in clinical treatment and in helpline contexts (Guillou-Landreat et al., 2016, Jones et al., 2025, Ronzitti et al., 2017). It has also been shown that persons with a prior gambling disorder diagnosis have significantly more mental health treatment contacts overall, particularly for in-patient care, whereas no significant difference was observed for out-patient contacts (Jones et al., 2025).

Suicidality can influence help-seeking behaviour for gambling problems in several ways. Suicidality can function as a barrier to help-seeking due to shame and stigma (Han et al., 2018, Marionneau and Nikkinen, 2022). Pronounced self-reliance, (perceived) lack of support and treatment services, price or excessive waiting times may function as further barriers to help-seeking (Bijker et al., 2022, Han et al., 2018). A prior Finnish study on completed suicides (Selin & Lind, 2024) found that social and economic stressors, as well as limited access to health and support services, increased the risk of gambling-related suicides.

On average, only one in five persons harmed by gambling problems seek any help (Bijker et al., 2022). Some prior evidence suggests that persons harmed by gambling problems and suicidality have lower levels of help-seeking than those experiencing suicidality without gambling problems (Séguin et al., 2010). However, studies show that for many, severe crises, such as a failed suicide attempt, can also function as a turning point to seek help (Hing et al., 2016, Marionneau and Nikkinen, 2022).

Research also shows that overall, individuals with suicidal ideation are more likely to use mental health services than those without suicidal ideation (Pirkis et al., 2001). Still, service use remains suboptimal, with only around half of adults with suicide-related experiences accessing mental health services. Potential barriers include self-reliance, uncertainty or fear with regard to care options, embarrassment and sociodemographic factors (Hom et al., 2015). In turn, facilitators to service use include informal support and low-threshold helplines (Batterham et al., 2022). However, there are also studies, that have shown that most people who have died by suicide have been in recent contact with healthcare services before their death (DelPozo-Banos et al., 2024, Partonen et al., 2022).

### 1.2 Context of this study

Understanding how help services reach those experiencing gambling-related suicidality is crucially important to developing better targeted interventions within this population and to removing potential barriers.

Finland is a well-suited context to investigate service use among individuals experiencing gambling problems and suicidality. Gambling is prevalent in Finland. According to the latest population study, 70 % of the population aged 15–74 years have gambled in the past year in 2023. Furthermore, 4.2 % engage in moderate-risk or problem gambling (Finnish Institute for Health and Welfare (THL), 2024). Finland counted 13.5 suicides per 100,000 inhabitants in 2023. Although suicide rates have declined in recent decades, suicide is the leading cause of death for those under 25 (Statistics Finland, 2024). In addition, one in ten Finnish adults reported suicidal thoughts in 2022 (Finnish Institute for Health and Welfare (THL), 2023).

Finland has a social and health service network to address both suicidality and gambling-related issues. Third sector organisations offer helpline and peer support services. In the public sector, local tax-funded wellbeing service counties are legally obliged to organise health and social services, including for gambling and mental health problems (Kangas and Kalliomaa-Puha, 2022, Karanikolos et al., 2024). In practice, gambling problems and co-occurring mental health problems (including suicidality) are addressed by outpatient addiction and psychiatric services or, in more severe situations, by outpatient and inpatient psychiatric hospitals. Social services offer support for social problems, including services for rehabilitation, housing, child welfare and financial wellbeing (Social Welfare Act 710/1982). Despite service availability, few persons harmed by gambling problems receive a gambling disorder diagnosis in Finland (Finnish Institute for Health and Welfare, 2025, Finnish Institute for Health and Welfare, 2025c, Salonen et al., 2022). Similarly, mental health problems continue to go unidentified before completed suicides (Partonen et al., 2024).

The primary aim of this study is to examine how gambling problems are associated with suicidality and service-use patterns among individuals who have engaged in gambling and experienced suicidal thoughts in the past year, using a Finnish population sample. Understanding these associations is critical for public health and clinical practice, as suicide is one of the most severe consequences of gambling-related harm, yet research on service use among persons with gambling and suicidality remains limited. By directly comparing service use among individuals who have gambled and experienced suicidal thoughts, the study seeks to distinguish between those who access services and those who do not. This knowledge is essential for identifying unmet needs, tailoring interventions, and ensuring that prevention and treatment services are responsive to those at highest risk.

## 2 Methods

### 2.1 Data and sample

Our data are derived from the population-based Healthy Finland survey, collected by the Finnish Institute for Health and Welfare from September 2022 to February 2023 (Sääksjärvi et al., 2024). The survey charts health, wellbeing and service use-related topics in the Finnish population. Using stratified random sampling, the survey was addressed to a nationally representative sample of 61,000 permanent Finnish residents (aged 20 + ). The survey was administered as paper and online versions in Finnish, Swedish, English, and Russian. In total, 28,153 individuals participated in the survey, resulting in a response rate of 46.3 %. The data were weighted based on age, gender, marital status, educational level, language, and area of residence. A more detailed data description is available elsewhere (Sääksjärvi et al., 2024). The dataset is large and includes a wide range of health-related variables in addition to gambling behaviour. While parts of this dataset have been used in several previous studies (e.g., Aalto et al., 2025, Sääksjärvi et al., 2025, Suominen et al., 2025) the specific associations examined in the current study — namely, the links between gambling problems, suicidality, and patterns of service use — have not been investigated using these data before. Only one earlier study using this dataset has focused on gambling, and it examined gambling severity, health-risk behaviours, and obesity (Grönroos et al., 2025). Present study therefore represents a novel application of the dataset to address this particular research question.

### 2.2 Measures

Education was classified into three groups (low, medium, high). Education was operationalized as years of education categorized into tertiles within each age and gender group to ensure comparability across different cohorts. While this approach does not directly indicate the highest completed education level, it broadly corresponds to the Finnish education system as follows: the lowest tertile (low) generally includes individuals with only compulsory basic education, the middle tertile (medium) corresponds roughly to upper secondary education, and the highest tertile (high) aligns with higher education such as universities or polytechnics. Education had 3.7 % of missing values. Sex and age had no missing values as information was retrieved from registers.

Household income was measured with the question: ‘How large was your household income last year (before tax)?’ The answer categories were under ‘€15,000′; ‘€15,001–35,000′; ‘€35,001–55,000′; ‘€55,001–75,000′; ‘over €75,001′. For the analysis, we combined the last two groups into ‘€55,001 or over’. Household income had 3.5 % of missing values.

Family status variable was created by combining information on the number of people and the number of under 18-year-olds in the household. The variable has three categories: single household, family without children, and family with children. Family status had a little higher percentage of missing values (6.0 %) compared to other sociodemographic factors.

Excessive alcohol use was assessed using the three-question *Alcohol Use Disorder Identification Test (AUDIT-C)*. The test measures alcohol consumption frequency, quantity and binge drinking, and it is scored 0–12 (Bush, 1998). *AUDIT-C* includes three standard questions: (1) frequency of drinking, (2) typical number of drinks consumed on drinking days, and (3) frequency of consuming six or more drinks on a single occasion. In the survey, respondents were asked to select the option that best reflects their current or typical drinking patterns, with examples provided for context. The response options followed the standard AUDIT-C categories, ranging from “never” to “four or more times per week” for drinking frequency, and from “1–2 drinks” to “10 or more drinks” for typical consumption. Thresholds for excessive alcohol use are ≥ 6 for men aged 20–64, ≥5 for women aged 20–64, and ≥ 4 for individuals aged 65 and older (Alcohol Problems. Current Care Guideline, 2015). The indicator had 2.8 % missing values.

Severe psychological distress was measured with the five-item *Mental Health Inventory questionnaire (MHI-5*) (Ware & Sherbourne, 1992). The questionnaire measures different feelings in the past four weeks using a 6-point scale. To calculate the final score, two items were reverse-coded, the scores were summed, and the raw scores were transformed to a scale ranging from 0 to 100. Scores (0–100) reflect overall mental health, with scores below 52 suggesting severe psychological distress. The indicator had 5.4 % missing values. Cronbach alpha for *MHI-5* was 0.85.

Suicidal thoughts was assessed by asking: ‘Have you had suicidal thoughts in the past 12 months?’ The response options were ‘no’ or ‘yes’. The question had 2.1 % missing values.

Long-term illness/health problem was assessed with the question*: ‘*Do you have any long-term illness or health problem? The response options were ‘no’ or ‘yes’. The question had 3.5 % missing values.

Loneliness was measured with the question: ‘Do you ever feel lonely?’ Response alternatives were ‘never’, ‘very rarely’, ‘sometimes’, ‘fairly often’, ‘all the time’. For our study, respondents who felt lonely at least ‘fairly often’ were categorised as lonely, and those who felt lonely ‘never’ or ‘very rarely’ as non-lonely. The indicator had 2.2 % missing values.

Gambling severity was measured with the *Problem Gambling Severity Index (PGSI)* (Ferris & Wynne, 2001). We used the following cut-offs: non-gambling, non-problem gambling (scored 0), low-risk (1–2), moderate-risk (3–7) and problem gambling (≥8). The first category (non-gambling) comprised of individuals who, in response to the question ‘Think about the past 12 months, how often did you gamble?’, reported that they had not gambled at all. The indicator had 4.1 % missing values. Cronbach alpha for *PGSI* was 0.90.

Health care use was measured with ‘Have you used health care services (e.g., doctor, nurse, hospital, dentist, dental hygienist) in the past 12 months?’ Social service use was measured with ‘Have you used social services in the past 12 months?’ Both questions could be answered ‘no’ or ‘yes’. These questions had 5.7 % and 5.4 % missing values respectively.

### 2.3 Statistical analyses

The data were analysed with IBM SPSS Statistics software (version 29.0.2.0) using complex sample design. We analysed sociodemographic factors and factors related to health and wellbeing in groups based on problem gambling severity. Statistical differences were examined using the χ^2^ test (Tables 1 & Supplementary file Table A). Logistic regression model was used to examine the associations between gambling severity and suicidal thoughts across different population subgroups. In this model, suicidal thoughts (yes/no) served as the dependent variable, while gambling severity and other covariates (e.g., age, gender, education, excessive alcohol use) were included as independent variables. Logistic regression is appropriate for this analysis because the outcome is binary (suicidal thoughts), allowing us to estimate odds ratios for the likelihood of reporting suicidal thoughts associated with each predictor. Table 2 presents the results of this model, stratified by population subgroups, highlighting how the presence and severity of gambling problems relate to suicidal thoughts while adjusting for potential confounders. This approach provides a clear and interpretable measure of association and enables comparison across different groups. Results are presented as odds ratios (ORs) with 95 % confidence intervals (CIs). In Model 1, only sociodemographic variables and gambling severity were adjusted for, whereas in Model 2, all potential confounding variables were included. The variables included for adjustment were selected based on prior literature and theoretical considerations, as they are known to be associated with both gambling problems and the outcomes of interest (e.g., suicidal thoughts). Adjusting for these covariates helps reduce potential confounding and allows us to better isolate the associations between gambling severity and the outcome.

**Table 1:** Excessive alcohol use, severe psychological distress, longstanding illness, loneliness and suicidal thoughts by PGSI categories (*%, N*).

	**PGSI categories**^a^										
	Problem gambling		Moderate-risk gambling		Low-risk gambling		Non-problem gambling		Non-gambling		P-value
**Measures**	%	N	%	N	%	N	%	N	%	N	
Excessive alcohol use	54.6	83	47.3	243	35.9	420	27.5	3,209	18.9	2,091	<0.001
Severe psychological distress	42.9	70	27.9	133	23.7	234	13.5	1,318	17.3	1,485	<0.001
Suicidal thoughts	31.3	44	15.6	56	12.0	110	6.5	541	8.3	597	<0.001
Long-term illness	57.2	105	59.7	380	59.0	816	56.6	7,746	52.8	7,733	<0.001
Loneliness	32.5	49	20.5	106	16.0	176	9.8	1,034	13.0	1,362	<0.001

a Problem gambling severity index (PGSI): non-gambling, non-problem gambling (score = 0), low-risk (1–2), moderate-risk (3–7), or problem gambling (≥8). Weighted based on age, gender, marital status, education level, language, and area of residence. The N-values are unweighted.

**Table 2:** Odds ratios (*ORs*) and 95 % confidence intervals (*CI*) for suicidal thoughts among all respondents (*N* = 28,153).

	**Model 1**		**Model 2**	
**Measures**	OR	95 % CI	OR	95 % CI
**Sex**				
Male	1.05	0.89–1.25	1.16	0.97–1.39
Female*	1	1	1	1
**Age**				
20–29	7.98	6.45–9.86	8.77	6.86–11.18
30–44	6.67	5.36–8.31	5.53	4.31–7.09
45–59	3.43	2.77–4.24	2.84	2.23–3.62
60+*	1	1	1	1
**Education**				
Low	1.06	0.87–1.30	0.94	0.75–1.17
Intermediate	0.90	0.72–1.11	0.87	0.69–1.09
High*	1	1	1	1
**Household income**				
15,000 or less	2.38	1.79–3.17	1.23	0.91–1.66
15,001–35,000	1.83	1.42–2.36	1.41	1.08–1.84
35,001–55,000	1.11	0.86–1.43	0.92	0.71–1.20
55,001 or more*	1	1	1	1
**Family status**				
Single household	1.74	1.29–2.34	1.19	0.87–1.62
Family without children	1.39	1.06–1.81	1.12	0.83–1.50
Family with children*	1	1	1	1
**PGSI**^a^**categories**				
Problem gambling	3.42	2.05–5.72	2.40	1.30–4.45
Moderate-risk gambling	1.99	1.26–3.16	1.48	0.91–2.41
Low-risk gambling	1.27	0.91–1.77	1.07	0.75–1.51
Non-problem gambling	0.90	0.75–1.08	0.93	0.77–1.30
Non-gambling*	1	1	1	1
**Excessive alcohol use**				
Yes	−	−	1.22	1.00–1.49
No*	−	−	1	1
**Severe psychological distress**				
Yes	−	−	5.87	4.83–7.12
No*	−	−	1	1
**Long-term illness**				
Yes	−	−	2.32	1.89–2.84
No*	−	−	1	1
**Loneliness**				
Yes	−	−	2.39	1.93–2.95
No*	−	−	1	1

a Problem gambling severity index (PGSI): non-gambling, non-problem gambling (score = 0), low-risk (1–2), moderate-risk (3–7), or problem gambling (≥8).

* Reference category. In model 1 sociodemographic and gambling severity are adjusted, whereas model 2, sociodemographic factors, gambling severity and factors related to health and wellbeing are adjusted.

After examining the association between gambling and suicidality we wanted to discover how people who experience suicidal thoughts use health care and social services. First, we examined health care and social service use among individuals with suicidal thoughts according to gambling severity using the χ^2^ test (Table 3) to discover whether the utilization of services is higher among individuals who gamble, and experience suicidal thoughts compared to non-gambling individuals with suicidal thoughts. All the gambling severity groups were compared to non-gambling group so four different tests were performed. Second, we wanted to distinguish between those who access services and those who do not by directly comparing service use among individuals who have gambled and experienced suicidal thoughts. Statistical differences in service utilization based on sociodemographic factors and factors related to health and welfare were examined using the χ^2^ −tests (Table 4).

**Table 3:** Utilization of health and social services among individuals with suicidal thoughts across gambling categories (*N* = 1405).

	**PGSI categories**^a^									
	Problem gambling		Moderate-risk gambling		Low-risk gambling		Non-problem gambling		Non-gambling	
**Measures**	%	N	%	N	%	N	%	N	%	N
Utilization of health care	96.1	40	82.8	49	82.3	96	88.4	471	81.8	497
χ^2^**-**test*	0.007		0.906		0.932		0.017		−	
Utilization of social services	37.2	14	10.2	5	22.5	24	15.7	75	15.7	103
χ^2^**-**test*	0.006		0.479		0.190		0.995		−	

a Problem gambling severity index (PGSI): non-gambling, non-problem gambling (score = 0), low-risk (1–2), moderate-risk (3–7), or problem gambling (≥8). Weighted based on age, gender, marital status, education level, language, and area of residence. The N-values are unweighted.

* All gambling severity groups were compared to non-gambling group (4 different test were performed).

**Table 4:** Factors that are related to utilization/non-utilization of health care and social services among individuals who had gambled and experienced suicidal thoughts in the past year (*N* = 756).

	Utilization of health care (*n* = 742)				Utilization of social services (*n* = 735)			
	**No**		**Yes**		**No**		**Yes**	
Measures	%	N	%	N	%	N	%	N
Sex								
Male	81.9	62	56.7	334	59.6	327	61.3	63
Female	18.1	19	43.3	327	40.4	289	38.7	56
χ^2^-test	<0.001				0.781			
Age								
20–29	42.3	23	24.7	112	28.2	116	20.6	17
30–44	27.3	18	38.6	192	34.6	160	50.7	48
45–59	24.6	26	25.2	184	25.8	174	20.6	32
60+	5.8	14	11.5	173	11.4	166	8.1	22
χ^2^-test	0.024				0.053			
Education								
Low	43.7	35	49.5	289	47.1	264	55.2	52
Intermediate	33.9	24	28.1	187	29.5	172	26.5	34
High	22.4	21	22.4	178	23.4	170	18.3	29
χ^2^-test	0.676				0.406			
Household income								
15,000 or less	33.9	23	21.1	122	20.4	108	32.6	34
15,001–35,000	32.6	29	32.2	209	31.0	191	38.4	46
35,001–55,000	11.4	11	19.9	152	20.4	141	13.1	21
55,001 or more	22.0	18	26.7	178	28.2	176	15.9	17
χ^2^-test	0.136				0.015			
Family status								
Single household	49.4	36	43.7	240	43.1	224	47.6	45
Family without children	43.5	34	42.4	286	47.2	292	23.8	29
Family with children	7.1	6	13.9	82	9.7	57	28.6	30
χ^2^-test	0.380				<0.001			
Excessive alcohol use								
Yes	54.7	34	36.3	220	39.4	212	33.7	35
No	45.3	47	63.7	439	60.6	403	66.3	83
χ^2^-test	0.011				0.382			
Severe psychological distress								
Yes	63.1	49	60.7	380	57.0	337	78.6	86
No	36.9	31	39.3	270	43.0	267	21.4	33
χ^2^-test	0.735				<0.001			
Long-term illness								
Yes	41.4	37	71.7	481	65.0	412	84.1	103
No	58.6	43	28.3	170	35.0	194	15.9	15
χ^2^-test	<0.001				0.003			
Loneliness								
Yes	42.1	33	60.8	240	37.1	218	47.7	55
No	57.9	47	39.2	413	62.9	391	52.3	63
χ^2^-test	0.701				0.018			

### 2.4 Ethics

The Ethics committee of the Finnish Institute for Health and Welfare approved the research protocol (THL/72/6.02.01/2022). All participants were provided with written information about the study. All participants gave informed consent to participate. All results are presented in a manner that does not allow identifying individual respondents.

## 3 Results

Problem gambling and gambling at moderate-risk levels were more common among men, younger individuals, those with lower education and income, and among single households (Supplementary Table A).

Over 50 % of persons harmed by problem gambling reported excessive alcohol use and a long-term illness. Among them, 43 % experienced severe psychological distress, 31 % had suicidal thoughts and 33 % had felt lonely (Table 2).

Suicidal thoughts were more common among individuals harmed by problem compared to individuals who did not gamble, even after accounting for sociodemographic factors (in Model 1), excessive alcohol use, severe psychological distress, loneliness, and long-term illness (in Model 2) (Table 2).

Of persons harmed by problem gambling and having suicidal thoughts, 96 % had used health services and 37 % had used social services in the past year (Table 3). In comparison, around 80 % of persons harmed by moderate or low-risk gambling and having suicidal thoughts had used health services, and less than 25 % had used social services. Overall, persons harmed by problem gambling and having suicidal thoughts were more likely to have used health care and social than non-gambling individuals with suicidal thoughts.

Individuals who had engaged in gambling and experienced suicidal thoughts in the past year but had not utilized health care services were more often 20–29 years old, male, and had excessive alcohol use (Table 4). They also did not report any long-term illnesses. By contrast, individuals with gambling involvement and suicidal thoughts who had utilized social services were more often 30–44 years old, had a household income of less than €35,001, and were from single households. Among this group, long-term illnesses and severe psychological distress were also common.

## 4 Discussion

This study has used population-level survey data to investigate the prevalence of suicidal thoughts among people experiencing gambling problems. We have also analysed the use of health care and social services and factors related to service use among individuals who have engaged in gambling and experienced suicidal thoughts. Our results shown that suicidal thoughts and other negative factors related to health and welfare are common among persons harmed by problem gambling: 31 % of persons harmed by problem gambling (PGSI score > 8) reported suicidal thoughts. In line with prior research, we also found overall higher levels of suicidal thoughts among persons harmed by problem gambling persisted after adjusting for other factors (Kristensen et al., 2024).

We found that 96 % of persons harmed by problem gambling and having suicidal thoughts had used some health services in the past year. Further, 37 % of them had also used social services. Individuals harmed by problem gambling and having suicidal thoughts used health and social services more frequently than non-gambling individuals with suicidal thoughts. Whilst our data do not allow us to determine whether help was sought for gambling-related suicidality specifically, our results clearly show that almost all persons harmed by problem gambling and having suicidal thoughts reported using health care during the past year and thus are clients of the health care system. Suicidality can function as an incentive for help-seeking (Hing et al., 2016; cf. Marionneau & Nikkinen, 2022).

We were also able to identify factors that were related to service and non-service use among those with suicidal thoughts and past year gambling. The present findings reveal distinct patterns in service utilization among individuals who had engaged in gambling and experienced suicidal thoughts. Young adults aged 20–29, male, individuals engaged in excessive alcohol use, and those who did not report any long-term illnesses were more likely to not have utilized any health care services during the past year. This suggests a critical gap in service provision for this group. While this may indicate unmet needs in these populations, our data do not allow us to determine whether lower utilization is driven by services being insufficient or inaccessible, or by lower help-seeking and service uptake even when services are available. Previous studies indicate that men and young adults are less likely to seek professional help for mental health problems, due to stigma, cultural norms, and perceptions of self-reliance (Galdas et al., 2005, Sheikh et al., 2025). Furthermore, the absence of chronic physical illnesses may reduce contact with the health care system, limiting opportunities for early identification and intervention. Excessive alcohol use, a known risk factor for both suicidal behaviour and gambling-related harm (Dowling et al., 2015, Lorains et al., 2011), may further hinder help-seeking and exacerbate vulnerability. These findings highlight the need for low-threshold, youth-oriented, and gender-sensitive interventions that address both gambling and alcohol-related vulnerability. Together, these findings underscore the importance of future research that can directly measure structural barriers, service accessibility, and group-specific help-seeking behaviour, ideally in larger samples where these mechanisms can be robustly tested.

In contrast, those who had utilized social services were more often individuals aged 30–44, living in single household and had a household income below €35,001. Interestingly, loneliness was less common in this group, suggesting that service engagement may confer protective effects. Cohabitation and social support have been shown to buffer against suicidal ideation and facilitate help-seeking (Kposowa, 2000), while social services can provide both financial and psychosocial support, mitigating the impact of gambling-related harm. These results underscore the importance of accessible social service and integrated service provision for adults facing socioeconomic disadvantage, as such approaches address multiple determinants of health simultaneously (Marmot et al., 2008).

Overall, our results yield three suggestions on how the health and social services should be improved to meet the needs of persons harmed by concurrent gambling problems and suicidal thoughts.

First, it must be ensured, that the health and social services professionals have sufficient training and tools to recognise gambling problems among those with suicidal ideation and vice versa, as well as to offer suitable treatment and support. The implementation of national care guidelines is one way to strengthen the competence of health and social services professionals. Our result echoes previous research showing that while persons harmed by gambling problems may not seek specialist services, they do access general health care (Roberts et al., 2019) − in our study, almost all of the participants with suicidal thoughts and severe gambling problems had been patients in health care during the past year. These results confirm that health care services continue having clients, who experience possibly fatal problem gambling, and it is possible (and probable) that gambling has not been recognized behind the service use.

In Finland, it has been found, that in the education programmes of basic social and health care degrees (such as a nurse’s degree), contents related neither gambling nor suicidality are included (Markkula et al., 2023). According to an international systematic review, general practitioners do not routinely screen for gambling problems, do not usually treat gambling disorder themselves and not more than every third is confident in their knowledge about gambling problems (Tatar et al., 2025). In Finland, gambling-related issues continue to be underdiagnosed in primary health care and hospitals (Finnish Institute for Health and Welfare, 2025, Finnish Institute for Health and Welfare, 2025c). Routine screening of gambling can face obstacles, including the need for health care professionals to focus on (other, more visible) immediate risks to health and wellbeing, perceiving problem gambling as a rare condition or lacking knowledge about problem gambling screening technics and referral pathways (Heath et al., 2025, Rodda et al., 2018).

Finland has Current Care Guidelines for both problem gambling (2023) and suicide prevention (2023), as well as a national programme for suicide prevention (2020). The suicide prevention programme in Finland includes six measures to improve the care services in the prevention of suicides by, for example, ensuring that the professionals have high-quality competence and shared, evidence-based models for addressing and assessing suicide risk as well as preventing suicides (Partonen, 2020). The Current Care Guideline for Problem Gambling (2023) includes systematic screening of suicide risk and protocols for assessment, while the Current Care Guideline for Suicide Prevention (2023) does not include gambling problems as a suicide risk factor. This discrepancy reflects a broader inconsistency in how different sectors understand and operationalise the links between gambling harm and suicidality. However, there are ongoing developments to connect these two in training programmes, and, for example, an online course for suicide prevention already includes contents related to gambling problems (Finnish Institute for Health and Welfare (THL), 2025a).

Effective implementation of these guidelines and improving the training of professionals within gambling problems and suicidality can advance the (early) detection of gambling-related suicidality. Further efforts are needed to ensure consistent implementation across all relevant sectors, and this may include, for example, embedding guideline-based screening protocols in electronic health records, ensuring that frontline staff (e.g., primary care) receive training on the guidelines, as well as developing local pathways that specify where to refer individuals presenting with both gambling problems and suicidality. Future updates to national guidelines are also needed, and these should aim to harmonise risk-assessment frameworks so that gambling is consistently recognised as a suicide risk factor.

Second, while the health care services are in a crucial role in recognition and treatment of gambling problems and suicidality, our results show also a higher frequency of social service use among those with gambling problems and suicidal thoughts compared to non-gamblers with suicidal thoughts. This suggests that the compound effect of these issues can increase the need for a range of services, such as social work or financial counselling. In this study 37 % of persons harmed by gambling problem and having suicidal thoughts reported of being clients of social services during the past year, while 14 % of Finnish population were estimated to be clients of social services in 2024 (Forsell et al., 2025). Financial problems have been connected to gambling-related suicidality and help-seeking (Marionneau & Nikkinen, 2022). However, social workers seem to have insufficient training and knowledge in gambling and be uncertain on how to support people experiencing gambling-related harm, and screening of gambling in social services is not yet a common practice (Bramley et al., 2019, Forward et al., 2022, Nower et al., 2023).

Thus, the role of social services in supporting people experiencing suicidality and problem gambling should be acknowledged. Gambling problems often cause financial and social problems, and financial, social, housing and employment services may be in support in the recovery in gambling problems in general. In preventing suicides, both social factors and mental illnesses are important to consider for example, lower socioeconomic position is associated with suicide (Li et al., 2011). The European Psychiatric Association (EPA) guidance on suicide treatment and prevention suggests that the treatment team for suicidality should include also social workers in addition to psychiatrists, for example (Wasserman et al., 2012).

Third, existing barriers to help-seeking need to be addressed. While the use of health care services was prevalent, the study also had 4 % of persons harmed by gambling with suicidal thoughts who had not reported of being patients in health care during the past year. Our findings showed that young, men and individuals using alcohol excessively with gambling problems and suicidal thoughts may remain outside formal support systems. Earlier research show that people who have died by suicide without receiving mental health services were likely to have diverse profiles: younger and older, male, from rural locations (Tang et al., 2022). Service practices need to be structured so that the services are easy to access, acknowledging individual service needs (related to, e.g., gender, age, financial situation or culture), the help-seekers are not sent back-and-forth and that they receive the treatment and support they need, both from health care and social services, as well as from other services, such as financial counselling or legal services. In Finland, fragmented service provision and long waiting times have been identified challenges in public health care provision. Recently, increased focus in Finland has been put on defining and setting ‘service paths’ for those requiring integrated services, for example, both social work and medical treatment (Karanikolos et al., 2024; Problem gambling, Current Care Guideline, 2023). As suicidality and gambling problems are often connected to a range of other social, health and financial issues, integrated service paths can form a critical component for treatment efforts.

### 4.1 Limitations and further studies

One limitation of our study concerns potential selection effects. Although the sample was weighted to improve representativeness, high non-response rates in population-based surveys may lead to underrepresentation of individuals with gambling problems, suicidal thoughts, mental health issues, or excessive alcohol use. Prior research suggests that people experiencing psychological distress, behavioural health problems, or social stigma are generally less likely to participate in surveys (Torvik et al., 2012). Consequently, our respondents may represent a somewhat healthier or more help-seeking segment of the population, potentially resulting in conservative estimates of both gambling-related harm and service non-use. Future studies should consider targeted recruitment strategies, mixed-mode data collection, or administrative data linkages to better capture high-risk or hard-to-reach groups (e.g. Kontto et al., 2025).

As our study relies on self-reported measures, our results are subject to recall and social desirability biases. We acknowledge that our study relied on self-reported data on utilization of health care and social services covering the past 12 months, which may be subject to recall bias and reporting inaccuracies. This limitation should be considered when interpreting the findings. An alternative approach involves using linked, routinely collected administrative data, which can provide more objective and longitudinal information on service use (Boering et al., 2025).

The cross-sectional design also limits our ability to draw causal conclusions between gambling, suicidality and service use. Longitudinal research is needed to better understand these relationships. Also, higher missing values (for PGSI 4.1 %, for MHI-5 5.4 %, health care use 5.4 % and for social service use 5.7 % of responses) in some of the variables may have introduced bias and impacted the generalisability of our findings. Further, loneliness was measured without specifying a time frame, which may affect interpretation. Finally, the lack of internationally standardised terminology for different forms of suicidality presents challenges for comparability across studies. Suicidal thoughts or suicidal ideation are not a clearly defined concepts, and subjective interpretations can range from fleeting thoughts to detailed plans. Further studies are also needed to address this complexity in measurement and interpretation.

We did not model potential interaction effects between measures, which could influence the estimated odds ratios. Future research could explore such interactions to provide a more nuanced understanding of these relationships. Further, we used logistic regression to estimate odds ratios rather than relative risks. While relative risks can be more intuitive and directly interpretable, particularly in population-based data, logistic regression is a well-established method for analysing binary outcomes and allows adjustment for multiple covariates. Conducting a relative risk analysis would require alternative modelling approaches, such as log-binomial regression with robust standard errors, which were beyond the scope of the current study. Future research could explore relative risks to complement the findings presented here.

To conclude, future studies should aim to better capture high-risk groups such as young adults, men, and individuals with gambling problems or excessive alcohol use, for example through targeted recruitment or mixed-mode data collection. Research should also explore barriers to service access and help-seeking, distinguishing between availability, accessibility, and personal factors. Larger or pooled samples would allow more robust subgroup analyses, and longitudinal designs could clarify causal relationships between gambling, suicidality, and service use. Finally, integrating survey data with administrative records could improve accuracy and reduce bias from non-response.

## 5 Conclusions

This study has used nationally representative survey data to study associations between gambling, suicidal thoughts and service use. We found that gambling problems and suicidal thoughts frequently co-occur. Persons harmed by concurrent problem gambling and suicidal thoughts also use health care services (96 %) and social services (37 %) at higher rates than those with suicidal thoughts but no gambling. Our findings have practical implications for social and health care providers. It is important to strengthen the training of social and health care professionals of co-occurring problem gambling and suicidality, improve screening for gambling problems, and work to reduce barriers to help via integrated support services, acknowledging the roles of both health and social services.

## 6 Funding sources

The study was funded by the Ministry of Social Affairs and Health, Finland, within the objectives of the §52 Appropriation of the Lotteries Act. Daily work of the authors TL, TG, MH, SC and KL at the Finnish Institute for Health and Welfare, was also funded by the Ministry. The Ministry had no role in the study design, analysis, or interpretation of the results of the manuscript or any phase of the publication process.

## CRediT authorship contribution statement

**Tiina Latvala:** Writing – review & editing, Writing – original draft, Project administration, Formal analysis, Conceptualization. **Maria Heiskanen:** Writing – review & editing, Writing – original draft, Data curation, Conceptualization. **Virve Marionneau:** Writing – review & editing, Writing – original draft. **Kalle Lind:** Writing – review & editing, Writing – original draft. **Tanja Grönroos:** Writing – review & editing, Writing – original draft. **Sari Castrén:** Writing – review & editing, Writing – original draft.

## Declaration of competing interest

The authors declare that they have no known competing financial interests or personal relationships that could have appeared to influence the work reported in this paper.

## Data Availability

The authors do not have permission to share data.
