# Supplementary material for: Suicidal thoughts, problem gambling severity and utilisation of health care and social services: A population-based study in Finland
Source: Addict Behav Rep. 2025 Dec 19;23:100658. doi: 10.1016/j.abrep.2025.100658 (PMC12813089; doi:10.1016/j.abrep.2025.100658)
Supplement: Supplementary Data 1 [file mmc1.docx]

**Supplementary Table A**. Sociodemographic factors by PGSI categories (*%*, *N*)

|  | PGSI categories^1^ | | | | | | | | | |  |
| --- | --- | --- | --- | --- | --- | --- | --- | --- | --- | --- | --- |
|  | Problem gambling | | Moderate-risk gambling | | Low-risk gambling | | Non-problem gambling | | Non-gambling | | P-value |
| Measures | % | N | % | N | % | N | % | N | % | N |  |
| Sex |  |  |  |  |  |  |  |  |  |  | <0.001 |
| Male | 1.7 | 110 | 3.9 | 400 | 7.0 | 775 | 50.5 | 5,927 | 37.0 | 4,589 |  |
| Female | 0.5 | 51 | 1.4 | 186 | 3.7 | 500 | 44.0 | 6,557 | 50.3 | 7,860 |  |
| Age |  |  |  |  |  |  |  |  |  |  | <0.001 |
| 20–29 | 1.9 | 24 | 2.9 | 45 | 7.9 | 122 | 31.2 | 550 | 56.1 | 964 |  |
| 30–44 | 1.6 | 46 | 3.3 | 96 | 5.6 | 200 | 46.6 | 1,748 | 43.0 | 1,392 |  |
| 45–59 | 1.2 | 41 | 2.6 | 125 | 4.9 | 241 | 58.0 | 2,856 | 33.2 | 1,607 |  |
| 60+ | 0.4 | 50 | 2.0 | 320 | 4.4 | 712 | 46.8 | 7,375 | 46.3 | 8,486 |  |
| Education |  |  |  |  |  |  |  |  |  |  | <0.001 |
| Low | 1.8 | 92 | 3.1 | 271 | 6.7 | 608 | 49.1 | 4,888 | 39.3 | 4,445 |  |
| Intermediate | 0.8 | 37 | 2.8 | 165 | 5.1 | 384 | 49.4 | 4,052 | 41.9 | 3,546 |  |
| High | 0.5 | 28 | 1.6 | 131 | 3.5 | 238 | 42.6 | 3,281 | 51.8 | 3,909 |  |
| Household income |  |  |  |  |  |  |  |  |  |  | <0.001 |
| 15,000 or less | 2.2 | 44 | 3.4 | 88 | 5.9 | 187 | 31.3 | 1,082 | 57.2 | 2,033 |  |
| 15,001–35,000 | 1.2 | 44 | 2.7 | 199 | 6.5 | 468 | 42.8 | 3,762 | 46.8 | 4,317 |  |
| 35,001–55,000 | 1.2 | 43 | 2.2 | 136 | 5.5 | 303 | 51.9 | 3,245 | 39.3 | 2,706 |  |
| 55,001 or more | 0.6 | 28 | 2.5 | 152 | 3.9 | 283 | 55.6 | 4,170 | 37.5 | 2,878 |  |
| Family status |  |  |  |  |  |  |  |  |  |  | <0.001 |
| Single household | 1.4 | 61 | 3.0 | 186 | 5.9 | 414 | 42.9 | 3,276 | 46.9 | 4,084 |  |
| Family without together | 0.9 | 70 | 2.5 | 320 | 5.3 | 689 | 48.2 | 7,284 | 43.1 | 6,877 |  |
| Family with children | 1.2 | 17 | 2.5 | 51 | 4.4 | 102 | 50.0 | 1,099 | 41.8 | 873 |  |

^1^Problem gambling severity index (PGSI): non-gambling, non-problem gambling (score = 0), low-risk (1–2), moderate-risk (3–7), or problem gambling (≥8). Weighted based on age, gender, marital status, education level, language, and area of residence. The N-values are unweighted.
